# The Relationship of Hedonic Hunger, Macronutrient Balance, Nutrition Knowledge, and Body Image and Weight Control with Dietary Intake in Student Athletes and Exercisers

**DOI:** 10.3390/nu16060772

**Published:** 2024-03-08

**Authors:** Amy Janiczak, Adrienne Forsyth, Xia Li, Gina Trakman

**Affiliations:** 1 Department of Sport, Exercise and Nutrition Studies, La Trobe University, Melbourne, VIC 3086, Australia; adrienne.forsyth@acu.edu.au (A.F.); g.trakman@latrobe.edu.au (G.T.); 2 School of Behavioural and Health Sciences, Australian Catholic University, Fitzroy, VIC 3065, Australia; 3 Department of Mathematics and Statistics, La Trobe University, Melbourne, VIC 3086, Australia; x.li2@latrobe.edu.au

**Keywords:** sport nutrition, dietetics, diet intake, training, physical activity

## Abstract

Dietary intake is known to impact athletic performance. The factors that influence dietary intake have been investigated widely, but their collective effect has not been examined. The primary aim of this cross-sectional study was to assess the relationship between dietary intake and nutrition knowledge, body image, weight control, macronutrient balance, and hedonic hunger. Forty-two student athletes or active individuals were recruited through contact with sporting organisations and course coordinators, advertising via twitter, and flyers posted within university buildings. Nutrition knowledge, body image, weight control, macronutrient balance, and hedonic hunger were measured using the Abridged Nutrition for Sport Knowledge Questionnaire, Body Image Disturbance Questionnaire and Contour Drawing Rating Scale, a Weight Fluctuation Measure, Australian Eating Score, and Power of Food Scale, respectively. Hierarchical regression analysis, correlation testing, and mean difference testing were applied. Carbohydrate intake, body image disturbance scores, weight fluctuation, and hedonic hunger for food tasted had a significant relationship (*R*^2^ = 64.6%, Adj *R*^2^ = 0.608%, *p* < 0.001) with dietary energy intake. Student athletes’ dietary intakes are influenced by multiple potentially modifiable factors. Future studies should use larger sample sizes, with interventions focusing on individual modifiable factors to determine how dietary intake can be most significantly impacted.

## 1. Introduction

Athletes commonly use nutrition strategies to improve athletic performance, enhance recovery from training, and achieve body composition changes that are perceived to provide performance benefits or desired for aesthetic reasons [1]. While dietary intake is known to have a multitude of benefits for athletic performance, athletes often fail to meet the recommended nutrition guidelines [2]. Therefore, it is relevant to understand the factors that impact athletes’ dietary intake. In this context, Birkenhead and Slater [3] proposed a theoretical framework to describe the determinants of food choice in athletes. Drawing on this framework and work done by Devlin [4], Trakman identified four key factors that professionals working with athletes could influence to change dietary behaviour: hedonic hunger, macronutrient balance, nutrition knowledge, and body image and weight control [5].

Nutrition knowledge is the cognitive process associated with the understanding of nutrition practices and recommendations; nutrition knowledge can be improved by health professionals via nutrition education interventions [6]. Body image is an individual’s perception and attitude towards their own body [7]. Several standalone interventions have been shown to improve body image, but the magnitude of the reported change is relatively small [8]. Macronutrient balance describes the relative contribution of carbohydrates, proteins, and fats to total energy intake. Modifying macronutrient balance, which can be achieved via education and advice from nutrition professionals, may impact ad libitum energy intake because of the disparate effects of specific macronutrients on gastric emptying, hunger, and satiety. For example, high-carbohydrate beverages induce faster gastric emptying than high-protein beverages [9], potentially extending satiety and thereby reducing total energy intake. Hedonic hunger is defined as “an ‘appetite’ for the pleasurable tastes of food” [3]. Hedonic hunger is distinguished from physiological hunger and appetite, which are largely dependent on the hormones leptin and ghrelin and may be harder for health professionals to impact. Weight loss programs using commercial meal replacement products have been shown to effectively reduce hedonic hunger [10,11].

Nutrition knowledge is arguably the most well-studied ‘modifiable factor’ of athletes’ dietary intake. When examining the relationship between athletes’ nutrition knowledge and dietary intake, several systematic literature reviews have identified significant, weak, and positive associations between higher nutrition knowledge and adherence to dietary recommendations [12,13,14], with nutrition education interventions found to improve dietary intake in relation to poor body image, and efforts to control body weight in athletes have often been related to the pressures to maintain body composition [15,16]. In sports that emphasise leanness or low body mass, athletes are more susceptible to disordered eating [17]. A review on weight loss practices in combat athletes found significant reductions in energy, carbohydrate, protein, fat, water, vitamin, and mineral intake prior to competition in three out of four studies [18]. Likewise, Jenner at al. [19] found that elite Australian football players often modify dietary intake, particularly to reduce energy and carbohydrate intake, prior to body composition assessments. Many studies that have explored nutrition knowledge in athletes have focused on student athletes [20,21,22,23,24,25,26], likely as this population is more widely accessible to researchers.

While both macronutrient balance and hedonic hunger have been identified as factors that health professionals can influence to modify dietary behaviour, limited research has investigated the impact of macronutrient balance on ad libitum dietary energy intake, with most research focusing on comparing macronutrient intake to sports nutrition guidelines [27,28] or aiming to determine the most advantageous amount and timing of macronutrients to assist with athletic goals [29,30,31,32,33,34,35,36]. Similarly, there is little research exploring the impact of hedonic hunger on athletes’ dietary intake; limited research to date has centred on the impact of exercise on hedonic hunger [37,38,39].

While the relationship between athletes’ dietary intake with nutrition knowledge and body image has previously been examined, there is a gap in our understanding of the impact of macronutrient balance and hedonic hunger on athletes’ food choices. Further, the collective effect of nutrition knowledge, body image, macronutrient balance, and hedonic hunger has never been explored. Accordingly, the primary aim of the present study is to measure the independent effects of nutrition knowledge, body image and weight control, macronutrient balance, and hedonic hunger on student athlete and exercisers’ dietary intake, and to explore the association between these factors. A secondary aim is to assess the association between measured variables and sociodemographic factors.

## 2. Methods

### 2.1. Ethics

This cross-sectional study was conducted according to the guidelines in the Declaration of Helsinki, and all procedures involving human subjects were approved by the La Trobe University Human Research Ethics Committee (HEC21041). Written informed consent was obtained from all subjects.

#### 2.1.1. Study Sample

Australian undergraduate university students, aged between 18 and 35 years of age, who were able to read and write in English, had access to the internet and a suitable electronic device, and who actively participated in exercise or sport for a minimum of 2.5 h per week were recruited to participate in an anonymous online survey from March to May 2021. Individuals who were pregnant and/or lactating were excluded, as these life stages have different dietary recommendations and could influence dietary intake and weight gain outside of the factors being investigated.

#### 2.1.2. Recruitment

Recruitment occurred via contact with sporting organisations and course coordinators, advertising via twitter, and flyers posted within university buildings.

### 2.2. Data Collection

Demographic data collected for this study consisted of 19 items adapted from previously created demographic questions used as part of the Nutrition for Sport Knowledge Questionnaire [40].

Body image was assessed using two scales: the Contour Drawing Rating Scale and Body Image Disturbance Questionnaire. The Contour Drawing Rating Scale contains four items reported on a 9-point scale related to the body type most closely resembling their current body type, what they believe the average body type of the opposite gender to be, and the ideal body types of themselves and the opposite gender [41]. The body image scores for the Contour Drawing Rating Scale were calculated as the difference between the current body type and the ideal body for the self and the opposite gender. The Body Image Disturbance Questionnaire measured body image disturbance with 12 items, 7 of which were multiple choice (coded one to five), and 5 of which were open-ended responses. The Body Image Disturbance Questionnaire score was calculated as a mean of the seven multiple-choice items. Individuals with a Body Image Disturbance Questionnaire score of three and above are considered to have high body image disturbance, and individuals with scores below three are considered to have normal body image disturbance levels [42,43].

A Weight Fluctuation Measure was developed by A Janiczak for the purposes of this study. This involved comparing the scale indicator of previous body type to the current body type reported in the Contour Drawing Rating Scale; the difference in body type was then converted to a score (−8 up to 8). This score indicates weight loss (negative score), weight gain (positive score), or a stable weight (zero score).

Hedonic hunger was measured using the Power of Food Scale [44], a 15-item survey validated for use in university students, which uses a five-point Likert scale. The total score was the average of all items. The Power of Food Scale contains three subscales, including the following: food available (*n* = 6), food present (*n* = 4), and food tasted (*n* = 5). The scores for these subscales were the mean of items related to those subscales. Higher scores indicate the greater power of food, which is indicative of greater hedonic hunger.

Nutrition knowledge was assessed using the Abridged Nutrition for Sport Knowledge Questionnaire developed by Trakman, Forsyth, Hoye, and Belski [40], which was previously validated for use in elite and recreational Australian athletes. The Abridged Nutrition for Sport Knowledge Questionnaire is comprised of 35 items made up of two sections: general nutrition knowledge (11) and sport nutrition knowledge (24). Abridged Nutrition for Sport Knowledge Questionnaire scores of 0–50% indicate poor nutrition knowledge, scores of 51–65% indicate average nutrition knowledge, scores of 66–74% indicate good nutrition knowledge, and scores of 75% or higher indicate excellent nutrition knowledge [45].

Macronutrient balance and dietary intake were assessed using the Australian Eating Survey [46], which is a validated food frequency questionnaire used to assess habitual dietary intake within the previous three to six months. The Australian Eating Survey consists of 15 demographic questions (for example, age, weight, height, gender, supplement use, eating and sitting behaviours) and 123 questions related to commonly consumed foods in the following subsections: drinks (*n* = 9), milk and dairy foods (*n* = 10), breads and cereals (*n* = 10), sweets and snacks (*n* = 12), main meals (*n* = 29), other foods (*n* = 17), vegetables (*n* = 24), fruit (*n* = 11), and miscellaneous (*n* = 1). As an incentive, participants were provided with a dietary analysis report upon completion of the Australian Eating Survey. The data provided to researchers included average grams of intake per macro and micronutrient (g/nutrient), percent of total energy intake (% of TEI) from macronutrients and food groups, and Australian Recommended Food Score details (possible scores between 0 and 73), as well as a record of the participant’s response to each question included in the Australian Eating Survey. Due to the nature of the cohort (i.e., active individuals), a tool for assessing diet quality as per healthy eating guidelines was used. An equivalent athlete-specific tool was not available at the time of data collection.

### 2.3. Data Analysis

#### 2.3.1. Missing Data

The Nutrition for Sport Knowledge Questionnaire accepted surveys with up to 11% of responses (nine questions) being missing values, a level of response that was previously established by Trakman [5] as acceptable. Only data from fully completed Australian Eating Surveys were included within the Australian Eating Survey dataset provided to researchers.

#### 2.3.2. Statistical Analysis

Data analysis was completed using statistical software (SPSS v27, IBM, Armonk, NY, USA). Normality testing was completed using the Kolmogorov–Smirnov test or the Shapiro–Wilk test (for samples above 50 and samples below 50, respectively). Additionally, visual inspection of normal Q-Q plots was performed. The visual inspection of boxplots was used to identify outliers, with the 5% trimmed mean checked to determine if the outliers present were having a large impact upon the mean of the value.

Differences between the scale variables in dichotomous demographic groups (e.g., male vs. female, or consuming supplements vs. not consuming supplements) were analysed using independent sample *t*-tests or Mann–Whitney U tests depending on the suitability of the analysis. In order to provide comparable results, Spearman’s rho correlation coefficient was used for all correlation tests (macronutrient intakes, energy intake, body image scores, nutrition knowledge scores, and hedonic hunger scores) within the crosstabulation. Hierarchical multiple linear regression analysis was completed on variables found to be significantly correlated through the Spearman’s correlation crosstabulation of complete data (records including AES information) because the significant variables were then able to be tested in a number of regression models [47]. As this study was exploratory in nature and there is no practical or conceptual model available, a hierarchical regression model was deemed appropriate for this purpose.

#### 2.3.3. Power Calculation

For the linear multiple regression analysis of factors that influence diet quality based on modelling six predictors (nutrition knowledge, Body Image Disturbance Questionnaire score, Power of Food Scale score, Contour Drawing Rating Scale scores, macronutrient balance, and weight fluctuation levels), 48 participants were required for a small effect size (d = 0.20), an alpha value of 0.05, and power of 80% [48].

## 3. Results

### 3.1. Participant Characteristics

In total, 111 participants (68.3% female) began the surveys for this study; 82 participants completed the demographics, Contour Drawing Rating Scale, Weight Fluctuation Scale, Power of Food Scale, Body Image Disturbance Questionnaire, and Abridged Nutrition for Sport Knowledge Questionnaire (completion rate: 74.5%), and 42 participants completed these surveys and the Australian Eating Survey (completion rate: 38.2%). Participant characteristics can be seen in Table 1.

### 3.2. Key Outcome Measures

The results for the Contour Drawing Rating Scale, Body Image Disturbance Questionnaire, Weight Fluctuation score, Power of Food Scale, and Abridged Nutrition for Sport Knowledge Questionnaire are provided below (Table 2).

Selected dietary intake results from the Australian Eating Survey are reported in Table 3. These variables have been selected due to their similarity to Acceptable Macronutrient Distribution Ratios [49] and athlete dietary recommendations [1].

### 3.3. Associations between Modifiable Factors

Crosstabulation of Spearman’s correlations demonstrates that there was a significant positive correlation between energy intake and fat per kg of body weight (*r_s_* = 0.78, *p* < 0.001), carbohydrate per kg of body weight (*r_s_* = 0.63, *p* = 0.000), protein per kg of body weight (*r_s_* = 0.71, *p* < 0.001), Weight Fluctuation score (*r_s_* = 0.39, *p* = 0.011), Power of Food Scale Food Tasted scale (*r_s_* = −0.35, *p* = 0.022), and Body Image Disturbance Questionnaire score (*r_s_* = −0.34, *p* = 0.028). There was a significant negative correlation between the Australian Recommended Food Score and the Power of Food Scale Food Present scale (*r_s_* = −0.31, *p* = 0.046). All other relationships explored were not statistically significant (*p* > 0.052). Information on all crosstabulation results is available in the Supplementary Materials.

### 3.4. Regression Analysis

Regression models were conducted using variables found to have significant correlations within Spearman’s correlation crosstabulation. Regression analyses included all participants who completed the Australian Eating Survey (*n* = 42). The regression model was statistically significant and indicated that 64.6% of the variance in kilojoule intake was due to Body Image Disturbance Questionnaire score, Weight Fluctuation score, Power of Food Scale Food Tasted scale, and carbohydrate per kg of body weight on kilojoule intake (megajoule intake with dietary fibre) (*R*^2^ = 0.646, Adj. *R*^2^ = 0.608, F(4, 37) = 16.90, *p* ≤ 0.001) (Table 4). The model was adjusted for the number of predictors within the model.

### 3.5. Differences in Outcome Measures Based on Socio-Demographic Factors

Males had significantly higher scores than females for energy intake with dietary fibre (mean rank males: 29.0; females: 19.45; *p* = 0.038) and current vs. ideal Contour Drawing Rating Scale score (median males: 0; females: −1; *p* = 0.005) (Supplementary Materials). Females had significantly higher scores than males in Body Image Disturbance Questionnaire mean (median males: 1.43; females: 2.14; *p* = 0.002) and hedonic hunger of Power of Food Scale Average score, Power of Food Scale Food Available, Power of Food Scale Food Present, and Power of Food Scale Food Tasted (mean male: 2.33, SD: 0.7; female: 3.03, SD: 0.76; *p* <0.001, median male: 1.833; female: 3.0; *p* = 0.004, mean rank male: 34.43; female: 53.67; *p* = 0.002, mean rank male: 31.0; female: 55.1, *p* < 0.001, respectively) (see Supplementary Materials).

Those with prior nutrition education had higher nutrition knowledge than those without prior nutrition education (% Total Nutrition Knowledge (nutrition education: 52.43; no nutrition education: 36.98; *p* = 0.005); %General Nutrition Knowledge (nutrition education: 53.45; no nutrition education: 40.25; *p* = 0.019); %Sport Nutrition Knowledge (nutrition education: 50.83; no nutrition education: 37.87; *p* = 0.019)). Likewise, those with prior nutrition education had a higher opposite average vs. Ideal Contour Drawing Rating Scale score than those without prior nutrition education (nutrition education: 1; no nutrition education: −1, *p* = 0.047) (see Supplementary Materials).

Participants who consumed supplements had higher opposite average vs. Ideal Contour Drawing Rating Scale scores than participants who did not consume supplements (Median: 0.0, IQR: 1 vs. median: −1.0, IQR: 1, *p* = 0.025). Likewise, supplement consumers had higher Australian Recommended Food Score extras than non-supplement consumers (mean rank 26.3 vs. 18.65, *p* = 0.031) (see Supplementary Materials).

Participants consuming a special diet had lower Australian Recommended Food Scores for meat consumption (*p* = 0.001), fat per kg of body weight (*p* = 0.033), protein per kg of body weight (*p* = 0.009), protein % of E (*p* < 0.001) and fat % of E (*p* = 0.001), and higher Australian Recommended Food Scores for meat alternatives (*p* < 0.0001) and carbohydrate % of E (*p* = 0.002) (see Supplementary Materials).

## 4. Discussion

The aims of this study were to (a) measure the relationship between nutrition knowledge, body image and weight control, macronutrient balance, hedonic hunger, and dietary intake in student athletes and exercisers; (b) assess the association between these factors in student athletes and exercisers; and (c) assess the differences in outcome measures based on socio-demographic factors. Regression analysis demonstrated that up to 64.6% of the difference in energy intake could be due to Body Image Disturbance Questionnaire scores, Weight Fluctuation scores, Power of Food Scale Food Tasted scores, and carbohydrate/kg of body weight. There were relatively few significant correlations between factors that professionals can influence and dietary intake variables, with no significant correlations found between diet quality and other outcome measures. It was found that males in this sample exhibited lower body image disturbance and hedonic hunger scores than females, while having a significantly higher kilojoule intake than females. Those with prior nutrition education had significantly higher nutrition knowledge scores than those without prior nutrition education. Individuals who consumed supplements were found to consume significantly more Australian Recommended Food Score extra foods than those who did not consume supplements, and those who followed a special diet consumed significantly less meat, protein, and fat and significantly more meat alternatives and carbohydrates.

### 4.1. Relationships of Modifiable Factors to Dietary Intake and Body Image Disturbance

It is expected based on previous research that body image disturbance would decrease energy intake [50], hedonic hunger would increase energy intake [51], and carbohydrate intake would increase with energy intake due to participant involvement in sport [1], as has been demonstrated in the present study. It would be beneficial to perform a similar analysis in a larger sample of athletes with more varied backgrounds. It is possible that other semi-modifiable factors (such as lifestyle and motives for participating in sport, fat-free mass, resting metabolic rate, hunger and appetite, taste and food preference, gastrointestinal discomfort, meal patterns, availability, and social facilitation [3,5]) and factors unlikely to be modified by sports health professionals (such as food cost, athlete income, and marketing) [5] may explain the remaining variance in intake; this possibility should be explored further in future studies.

### 4.2. Associations between Modifiable Factors

The correlation between energy intake and fat, carbohydrate, and protein intake was expected as macronutrients are components of dietary intake. The positive significant correlation between energy intake and Weight Fluctuation score could be related to dieting behaviours, as weight loss often coincides with energy deficits [52], and weight gain coincides with energy surplus. Likewise, the negative significant correlation between energy intake and the Power of Food Scale subsection “Food Tasted” may be because the Power of Food Scale is a measure of urge to eat rather than the actual consumption of food [53], and the urge to eat may be ignored within athlete or active populations who do not compensate for energy expended during exercise [3]. It is also likely that individuals who are consciously restricting energy intake for the purpose of weight loss may ignore the urge to eat due to dietary choices in line with their diet goals [54]. Similarly, the significant negative correlation of energy intake with Body Image Disturbance Questionnaire score may be because individuals with high body image disturbance scores are more likely to exhibit disordered eating behaviours [43] and may restrict dietary intake. This relationship may also be related to the type of sport athletes participate in, for example, higher rates of eating disorders have been demonstrated with body building [16], professional jockey [55], female tennis [56], and swimming [57] athletes. Further investigation of the difference in energy intake between athlete compensators and non-compensators in relation to these factors would be of interest.

Of note, the Australian Recommended Food Score food quality index’s significant negative correlation with the Power of Food Scale Food Present subscale indicates that the influence of food present within the immediate environment negatively impacts the quality of the food consumed. This may have been influenced by dietary changes due to a change in food environment with increased access to discretionary foods [58] caused by individuals spending more time in the home because of COVID-19 restrictions around working and recreation.

### 4.3. Differences in Outcome Measures Based on Socio-Demographic Factors

Hedonic hunger and Body Image Disturbance were higher in females than males, with current vs. ideal Contour Drawing Rating Scale score and kilojoule intake with dietary fibre being higher in males than females. Energy intake may be expected to be greater for men than for women due to the higher energy requirements of men [59]. The differences in Contour Drawing Rating Scale and Body Image Disturbance Questionnaire scores may be due to a higher rate of body image disturbance in women than men. It has previously been found that college women had a higher rate of body image disturbance than men [42,60], with heavier women having greater body image disturbance than lighter women, and white women having greater body image disturbance than African American women [60].

In the present study, nutrition education was associated with better nutrition knowledge. These findings are in line with a recent systematic literature review which reported that nutrition education interventions improved nutrition knowledge in randomised controlled trials (*n* = 13) and single-arm pre/post-design (*n* = 19) studies [6]. Similar results were found in a recent systematic literature review by Janiczak et al. [61], which showed all studies (10 out of 24 included) that used nutrition education interventions positively impacted nutrition knowledge, with eight of those studies having a positive impact on some aspect of dietary intake. Interestingly, we also found that nutrition education was associated with improved body contour ratings, indicating that nutrition education may have a role to play in improving body image.

The Australian Eating Survey results showed significant differences in nutrient intake amongst those who followed special diets, which was expected due to differences in food group intake related to specialised dietary requirements [46]. Due to the large number of vegetarian/vegan type diets that are featured as special diets in this population, these differences are axiomatic.

### 4.4. Limitations

The sample size for this study was relatively small. The population used within this study (Australian undergraduate university students) is not representative of the general population or the athlete population. The population of this study may have a higher nutrition knowledge than non-university-educated populations. Future studies exploring this topic may wish to include education level as an additional variable in a multiple linear regression model in order to control the effect of education on nutrition knowledge or recruit non-university-educated athletes, as this is an understudied population.

Increased levels of at-home study and work due to the COVID-19 pandemic may have had an impact on the food environment by increasing food availability and possibly limiting access to different types of foods due to shortages, which could further influence levels of hedonic hunger, dietary intake and macronutrient balance, and weight fluctuation within this period [62]. If weight did fluctuate during this time, it is possible that body image has also been impacted by this.

The tool used for measuring weight fluctuation within this study was not validated for use. As such, it is possible that the results for weight fluctuation may be biased. The use of the subjective measure of the Contour Drawing Rating Scale to measure weight fluctuation cannot provide a specific weight difference. Therefore, there is a need to validate this tool prior to use in future studies to ensure that accurate results are obtained.

Statistical significance was assessed within this study rather than mean difference between the two groups and the imprecision of the difference. The use of the regression model has been criticised for its lack of generalisability with a new sample of data [63]. However, the exploratory nature of this study required the use of a hierarchical regression model as an initial method of modelling [47]. Future studies may use more established models with larger sample sizes for more generalisability.

### 4.5. Strengths

To our knowledge, one other study [64] has assessed multiple factors that influence dietary intake in student athletes/exercisers. Thurecht and Pelly’s [64] paper included a wide variety of factors, including some that are not easily modifiable by nutrition/dietetics professionals. Therefore, the focus of the current study on modifiable factors that may influence dietary intake is novel. The tools used in this study are appropriate to the population as they have been validated with university students [42,44,60], athletes [40], or Australian healthy adult populations [46].

## 5. Conclusions

The results of this study provide support for a relationship between dietary intake and macronutrient balance, hedonic hunger, and weight control and body image in student athletes and exercisers. In turn, these factors are associated with a variety of socio-demographic factors. These results provide initial evidence that future interventions aiming to modify athletes’ dietary intake should focus on factors beyond nutrition knowledge.

## Figures and Tables

**Table 1:** Participant characteristics.

Variable	Part 1 *n* = 82	Part 1 & 2 *n* = 42
Gender		
- Male (*n*, %)	26 (31.7%)	9 (21.4%)
- Female (*n*, %)	56 (68.3%)	33 (78.6%)
Age (years) Median (IQR)	21.0 (3)	21.0 (3)
Plays competitive sport		
- Yes (*n*, %)	53 (64.6%)	27 (64.3%)
- No (*n*, %)	29 (35.4%)	15 (35.7%)
Sport type (*n*)	*n* = 53 AFL (5) Basketball (3) Cycling (2) Hockey (2) Endurance running (5) Soccer (football) (7) Swimming (1) Triathlon (1) Netball (5) Boxing (1) Weightlifting (5) Martial arts (2) Touch football (1) Other (13) Missing (29)	*n* = 27 AFL (2) Basketball (2) Cycling (2) Hockey (1) Endurance running (4) Soccer (football) (3) Triathlon (1) Netball (1) Boxing (1) Weightlifting (1) Martial arts (1) Other (8) Missing (15)
Highest level of sport	*n* = 53	*n* = 27
- Local (*n*, %)	33 (62.3%)	14 (51.9%)
- State (*n*, %)	10 (18.9%)	7 (25.9%)
- National (*n*, %)	6 (11.3%)	3 (11.1%)
- International (*n*, %)	4 (7.5%)	3 (11.1%)
Nutrition education *		
- Yes (*n*, %)	29 (35.4%)	15 (35.7%)
- No (*n*, %)	53 (64.6%)	27 (64.3%)
Nutrition student		
- Yes (*n*, %)	15 (18.3%)	7 (16.7%)
- No (*n*, %)	67 (81.7%)	35 (83.3%)
Special diet		
- Yes (*n*, %)	54 (65.9%)	25 (59.5%)
- No (*n*, %)	28 (34.1%)	17 (40.5%)
Special diet types (*n*)	*n* = 28	*n* = 25
	Vegan (7)	Vegan (6)
	Vegetarian (13)	Vegetarian (7)
	Pescatarian (3)	Pescatarian (1)
	Low FODMAP (1)	Low FODMAP (1)
	Gluten free (4)	Gluten free (2)
	Intermittent fasting (2)	Intermittent fasting (1)
	Other (5)	Other (5)
Supplement intake		
- Yes (*n*, %)	34 (41.5%)	15 (35.7%)
- No (*n*, %)	48 (58.5%)	27 (64.3%)
Supplement types (*n*)	*n* = 34 Multivitamin (7) Protein supplement (14) Beta alanine (3) Creatine (7) Probiotic (3) Omega 3 (3) Fish Oil (5) Zinc (4) Magnesium (13) Iron (16) Calcium (1) Vitamin D (7) Vitamin C (3) B vitamins (4) Folate (1) Collagen (2) Fibre (1) Lysine (1) Maca (1) Tyrosine (1)	*n* = 15 Multivitamin (2) Protein supplement (5) Beta alanine (2) Creatine (3) Probiotic (2) Omega 3 (1) Fish Oil (1) Magnesium (6) Iron (10) Calcium (1) Vitamin D (4) Vitamin C (2) B vitamins (3) Folate (1) Collagen (2) Lysine (1)

Notes Sports were categorised with 20 predetermined categories, one of which was ‘other’, allowing participants to specify a sport that was not included within the list (such as ballet, cross-country skiing, or gymnastics). * Nutrition education was classified as formal studies in human nutrition, which may include a university subject, university course, a specialised course, an online course, or diploma.

**Table 2:** Results across outcome measures.

Variable	Part 1 (*n* = 82)	Part 1 & 2 (*n* = 42)
ANSKQ		
- %TNK (mean (SD))	55.12 (12.29)	55.85 (13.27)
- %GNK (median (IQR))	72.73 (27.27)	72.73 (27.27)
- %SNK (mean (SD))	49.44 (14.93)	50.10 (16.32)
CDRS (median (IQR))		
- Current vs. ideal	−1.0 (2)	−1.0 (2)
- Opposite average vs. ideal	−1.0 (1)	−1.0 (1)
BIDQ (median (IQR))	2.0 (1.43)	2.14 (1.57)
Weight Fluctuation (median (IQR))	0.0 (2)	0.0 (1)
PFS		
- Average (1–5) (mean (SD))	2.83 (0.81)	2.79 (0.80)
- Food available (1–5) (median (IQR))	2.75 (1.71)	2.58 (2.0)
- Food present (1–5) (median (IQR))	2.88 (1.75)	2.50 (2.06)
- Food tasted (1–5) (median (IQR), mean (SD)))	2.9 (1.05)	2.96 (0.85)

Note. ANSKQ, Abridged Nutrition for Sport Knowledge Questionnaire score. %TNK, percent of Total Nutrition Knowledge questions correct. %GNK, percent of General Nutrition Knowledge questions correct. %SNK, percent of Sport Nutrition Knowledge questions correct. CDRS, Contour Drawing Rating Scale score. BIDQ, Body Image Disturbance Questionnaire score. PFS, Power of Food Scale.

**Table 3:** Average results from the Australian Eating Survey (*n* = 42).

Variable	Mean (SD), Range
ARFS	38.64 (9.03) 19, 56
KJ with DF	9208.29 (2587.25) 4268.29, 16873.95
CHO/kg BW (g)	4.05 (1.43) 0.91, 7.96
Fat/kg BW (g)	1.23 (0.36) 0.55, 1.97
PRO/kg BW (g)	1.35 (0.47) 0.63, 2.59
CHO% of E Median (IQR) Range	48.07 (12) 24, 61
Fat% of E Median (IQR) Range	34.05 (9) 25, 53
PRO% of E	16.29 (3.92) 10, 27

Note. ARFS, Australian Recommended Food Score. KJ with DF, kilojoule intake with dietary fibre. CHO/kg BW, carbohydrate intake per kilogram of body weight. Fat/kg BW, fat intake per kilogram of body weight. PRO/kg BW, protein intake per kilogram of body weight. g, grams. CHO% of E, carbohydrate percentage of total energy intake. Fat% of E, fat percentage of total energy intake. PRO% of E, protein percentage of total energy intake.

**Table 4:** Hierarchical regression analysis examining megajoule intake with BIDQ score, Weight Fluctuation score, PFS Food Tasted scale, and carbohydrate/kg/body weight.

Variable	Megajoule Intake with Dietary Fibre							
	Model 1		Model 2		Model 3		Model 4	
	Β	95% CI	Β	95% CI	Β	95% CI	Β	95% CI
Intercept	4.353 **	2.49–6.22	6.729 **	4.26–9.12	6.946 **	4.61–9.28	9.190 **	6.27–12.11
CHO/kg	1.20 **	0.77–1.63	1.123 **	0.72–1.53	1.058 **	0.67–1.45	0.984 **	0.61–1.36
BIDQ			−0.915 *	−1.59–−0.24	−0.918 *	−1.56–−0.28	−0.834 *	−1.44–−0.23
WFS					0.745	0.13–1.36	0.730	−0.15–1.31
PFS food Tasted							−0.719	−1.34–−0.10
*R* ^2^	0.438		0.528		0.593		0.646	
Adj *R*^2^	0.424		0.503		0.560		0.608	
Δ*R*^2^	0.438		0.09		0.065		0.053	
*p* value	<0.001		<0.001		<0.001		<0.001	

Note. *n* = 42. * *p* < 0.05, ** *p* < 0.001. CHO/kg, carbohydrate per kilogram of body weight. BIDQ, Body Image Disturbance Questionnaire score. WFS, Weight Fluctuation score. PFS, Power of Food Scale.

## Data Availability

Data is contained within the article and Supplementary Materials.

## Supplementary Materials

The following supporting information can be downloaded at: https://www.mdpi.com/article/10.3390/nu16060772/s1, Table S1: Spearman’s Crosstabulation results for main outcomes (*n* = 42); Table S2: Differences in outcome measure based on gender (*n* = 82); Table S3: Differences in outcome measure based on prior nutrition education (*n* = 82); Table S4: Differences in outcome measure based on supplement intake; Table S5: Differences in outcome measure based on consumption of a special diet (*n* = 42).
