# Supplementary material for: The Relationship of Hedonic Hunger, Macronutrient Balance, Nutrition Knowledge, and Body Image and Weight Control with Dietary Intake in Student Athletes and Exercisers
Source: Nutrients. 2024 Mar 8;16(6):772. doi: 10.3390/nu16060772 (PMC10974079; doi:10.3390/nu16060772)
Supplement: Supplementary file 1 [file nutrients-16-00772-s001.zip › nutrients-2877756-supplementary.pdf]

Supplementary Material

Table S1: Spearman's Crosstabulation results for main outcomes (n=42)

	WC CDRS	PFS FT	BIDQ	MJDF	CvsI CDRS	OAvsI CDRS	PFS Total	PFS FA	PFS FP	%TNK	%GNK	%SNK	ARFS	Fat/kg/bw	CHO/kg/bw	PRO/kg/bw	PRO% of E	CHO% of E	Fats% of E
WC CDRS	1																		
PFS FT	-0.120	1																	
	0.450																		
BIDQ	-0.042	0.206	1																
	0.793	0.191																	
MJDF	.387*	-.353*	-.340*	1															
	0.011	0.022	0.028																
CvsI CDRS	-0.140	-0.112	-.441**	-0.124	1														
	0.376	0.478	0.003	0.434															
OAvsI CDRS	0.007	.359*	-0.096	-0.069	0.105	1													
	0.965	0.020	0.546	0.663	0.510														
PFS Total	0.020	.743**	.388*	-0.185	-0.278	0.245	1												
	0.900	0.000	0.011	0.241	0.075	0.118													
PFS FA	0.090	.527**	.428**	-0.093	-0.239	0.291	.864**	1											
	0.570	0.000	0.005	0.558	0.128	0.061	0.000												
PFS FP	-0.111	.512**	0.301	-0.182	-.350*	0.062	.809**	.507**	1										
	0.483	0.001	0.052	0.248	0.023	0.696	0.000	0.001											
%TNK	-0.276	-0.115	-0.105	-0.131	0.222	-0.103	-0.301	-0.150	-.422**	1									
	0.077	0.467	0.507	0.408	0.157	0.515	0.053	0.343	0.005										
%GNK	0.004	-0.163	0.012	-0.061	-0.079	-0.271	-0.118	-0.034	-0.175	.581**	1								
	0.980	0.301	0.940	0.703	0.620	0.082	0.456	0.832	0.269	0.000									
%SNK	-.326*	-0.050	-0.082	-0.146	0.287	-0.020	-0.293	-0.150	-.415**	.900**	0.194	1							
	0.035	0.751	0.607	0.356	0.066	0.901	0.060	0.342	0.006	0.000	0.219								
ARFS	0.179	-0.173	-0.026	0.191	-0.011	0.014	-0.240	-0.155	-.309*	0.240	0.208	0.195	1						
	0.258	0.274	0.871	0.226	0.943	0.931	0.126	0.327	0.046	0.126	0.185	0.215							
Fat/kg/bw	0.192	-0.107	-0.303	.778**	-0.030	0.148	-0.030	-0.024	-0.046	-0.156	-0.158	-0.133	0.145	1					
	0.224	0.499	0.051	0.000	0.851	0.350	0.853	0.882	0.772	0.324	0.317	0.400	0.361						
CHO/kg/bw	0.147	-0.198	-0.128	.629**	-0.025	0.006	-0.181	-0.123	-0.232	0.052	-0.023	0.052	0.256	.512**	1				
	0.352	0.208	0.418	0.000	0.874	0.971	0.252	0.437	0.139	0.743	0.883	0.745	0.101	0.001					
PRO/kg/bw	0.234	-0.119	-0.220	.708**	-0.049	0.180	-0.054	0.013	-0.081	-0.066	-0.204	-0.007	0.205	.811**	.449**	1			
	0.136	0.455	0.162	0.000	0.757	0.254	0.732	0.935	0.608	0.677	0.196	0.965	0.192	0.000	0.003				
PRO% of E	0.031	0.034	-0.108	0.059	0.010	0.076	0.037	0.094	0.062	-0.044	-0.142	0.005	-0.054	0.190	-.408**	.539**	1		
	0.845	0.829	0.495	0.712	0.952	0.632	0.818	0.555	0.695	0.783	0.371	0.977	0.732	0.227	0.007	0.000			
	-0.075	0.000	0.128	-0.065	0.027	-0.120	-0.082	-0.069	-0.139	0.193	0.093	0.178	0.013	-0.300	.565**	-.306*	-.733**	1	

CHO% of E	0.635	1.000	0.420	0.684	0.863	0.449	0.607	0.665	0.380	0.220	0.559	0.260	0.933	0.053	0.000	0.049	0.000		
Fats% of E	0.050	0.053	-0.167	0.145	-0.018	0.161	0.141	0.083	0.216	-0.297	-0.142	-0.291	-0.170	.453**	-.430**	0.238	.501**	-.878**	1
	0.751	0.738	0.290	0.359	0.911	0.307	0.374	0.600	0.169	0.056	0.370	0.062	0.283	0.003	0.004	0.129	0.001	0.000	

*Note. WC CDRS, Weight Change Contour Drawing Rating Scale score. PFS FT, Power of Food Scale Food Tasted Subscale score. BIDQ, Body Image Disturbance Questionnaire score. MJDF, Megajoules of energy intake with dietary fibre. CvsI CDRS, Current versus Ideal Contour Drawing Rating Scale score. OAvsI CDRS, Opposite Average versus Ideal Contour Drawing Rating Scale score. PFS Total, Power of Food Scale Total score. PFS FA, Power of Food Scale Food Available Subscale score. PFS FP, Power of Food Scale Food Present Subscale score. %TNK, Percent score of Total Nutrition Knowledge. %GNK, Percent score of General Nutrition Knowledge. %SNK, Percent score of Sport Nutrition Knowledge. ARFS, Australian Recommended Food Score. Fat/kg/bw, Fat intake grams per kilogram of body weight. CHO/kg/bw, Carbohydrate intake grams per kilogram of body weight. PRO/kg/bw, Protein intake grams per kilogram of body weight. PRO% of E, Protein percentage of total energy intake. CHO% of E, Carbohydrate percentage of total energy intake. Fats% of E, Fat percentage of total energy intake. *, $p<0.05$. **, $p<0.01$.*

Table S2. Differences in outcome measure based on gender (n=82).

	Male Scores	Female Scores	Test result
Energy intake with dietary fibre (n=42) Mean rank	29.0	19.45	U=81.0, z=-2.069, p=0.038 (exact sig)
Current vs. Ideal CDRS score Median (IQR)	0(1)	-1(2)	U = 629.5, z = -2.818, p=0.005
BIDQ Median (IQR)	1.43(0.79)	2.14(1.36)	U=1198.5, z=3.071, p=0.002
PFS Mean	2.33 (0.7)	3.03(0.76)	t (93) = -4.177, p <0.0001
PFS Food Available Median (IQR)	1.833 (1.67)	3.0(1.33)	U=1293.0, z=2.904, p=0.004
PFS Food Present Mean rank	34.43	53.67	U=1318.0, z=3.113, p=0.002
PFS Food Tasted Mean rank	31.0	55.1	U=1414.0, z=3.903, p<0.0001

Note. CDRS, Contour Drawing Rating Scale. BIDQ, Body Image Disturbance Questionnaire score. PFS, Power of Food Scale score. IQR, interquartile range.

Table S3. Differences in outcome measure based on prior nutrition education (n=82).

	Prior nutrition education group scores	No prior nutrition education group scores	Test result
%TNK Mean rank	52.43	36.98	U=512.0, z=-2.792, p=0.47
%GNK Mean rank	53.45	40.25	U=641.5, z=-2.349, p-0.019
%SNK Mean rank	50.83	37.87	U=560.0, z=-2.343, p=0.019
Opposite average vs. Ideal CDRS score Median (IQR)	1(1)	-1(1)	U=1295.5, z=1.988, p=0.47

Note. %TNK, percentage of total nutrition knowledge questions correct. %GNK, percentage of general nutrition knowledge questions correct. %SNK, percentage of sport nutrition knowledge questions correct. CDRS, Contour Drawing Rating Scale. IQR, interquartile range.

Table S4. Differences in outcome measure based on supplement intake.

	Those who consume supplement group score	Those who do not consume supplements group score	Test result
ARFS extras consumption (=42) Mean rank	26.63	18.65	U=279.5, z=2.156, p=0.031
Opposite average vs. Ideal CDRS score (n=82) Median (IQR)	0.0 (1)	-1.0 (1)	U=1417.0, z=2.246, p=0.025

Note. ARFS, Australian Recommended Food Score. CDRS, Contour Drawing Rating Scale. IQR, interquartile range.

Table S5. Differences in outcome measure based on consumption of a special diet (n=42).

	Those who consume a special diet group score	Those who do not consume a special diet group score	Test result
ARFS meat consumption Mean rank	14.0	26.6	U=85.0, z=-3.378, p=0.001
ARFS meat alternatives Mean rank	30.38	15.46	U=363.5, z=3.910, p<0.0001
fat/kg/bw Mean (SD)	1.09 (0.29)	1.33(0.38)	t(40)=2.211, p=0.33 [b]
PRO/kg/bw Mean rank	15.71	25.44	U=114.0, z=-2.524, p=0.012
PRO% of E Mean rank	13.32	27.06	U=73.5, z=-3.579, p<0.0001
Fat% of E Mean (SD)	30.65 (4.17)	36.36(5.99)	t(40)=3.405, p=0.002
CHO% of E Median (IQR)	56 (12)	47(10)	U=331.0, z=3.044, p=0.002

Note. ARFS, Australian Recommended Food Score. Fat/kg/bw, fat intake per kilogram of body weight. PRO/kg/bw, protein intake per kilogram of body weight. PRO% of E, protein percentage of total energy intake. Fat% of E, fat percentage of total energy intake. CHO% of E, carbohydrate percentage of total energy intake.
